# Laboratory Stock Variants of the Archetype Silver Resistance Plasmid pMG101 Demonstrate Plasmid Fusion, Loss of Transmissibility, and Transposition of Tn*7*/*pco*/*sil* Into the Host Chromosome

**DOI:** 10.3389/fmicb.2021.723322

**Published:** 2021-08-19

**Authors:** Steven P.T. Hooton, Alexander C.W. Pritchard, Karishma Asiani, Charlotte J. Gray-Hammerton, Dov J. Stekel, Lisa C. Crossman, Andrew D. Millard, Jon L. Hobman

**Affiliations:** ^1^School of Biosciences, University of Nottingham, Sutton Bonington Campus, Sutton Bonington, United Kingdom; ^2^Department of Genetics and Genome Biology, University of Leicester, Leicester, United Kingdom; ^3^Sequenceanalysis.Co.uk, Innovation Centre, Norwich, United Kingdom; ^4^School of Biological Sciences, University of East Anglia, Norwich, United Kingdom

**Keywords:** plasmid, silver resistance, pMG101, PacBio, recombination

## Abstract

*Salmonella* Typhimurium carrying the multidrug resistance (MDR) plasmid pMG101 was isolated from three burns patients in Boston United States in 1973. pMG101 was transferrable into other *Salmonella* spp. and *Escherichia coli* hosts and carried what was a novel and unusual combination of AMR genes and silver resistance. Previously published short-read DNA sequence of pMG101 showed that it was a 183.5Kb IncHI plasmid, where a Tn*7*-mediated transposition of *pco*/*sil* resistance genes into the chromosome of the *E. coli* K-12 J53 host strain had occurred. We noticed differences in streptomycin resistance and plasmid size between two stocks of *E. coli* K-12 J53 pMG101 we possessed, which had been obtained from two different laboratories (pMG101-A and pMG101-B). Long-read sequencing (PacBio) of the two strains unexpectedly revealed plasmid and chromosomal rearrangements in both. pMG101-A is a non-transmissible 383Kb closed-circular plasmid consisting of an IncHI2 plasmid sequence fused to an IncFI/FIIA plasmid. pMG101-B is a mobile closed-circular 154 Kb IncFI/FIIA plasmid. Sequence identity of pMG101-B with the fused IncFI/IncFIIA region of pMG101-A was >99%. Assembled host sequence reads of pMG101-B showed Tn*7*-mediated transposition of *pco*/*sil* into the *E. coli* J53 chromosome between *yhiM* and *yhiN*. Long read sequence data in combination with laboratory experiments have demonstrated large scale changes in pMG101. Loss of conjugation function and movement of resistance genes into the chromosome suggest that even under long-term laboratory storage, mobile genetic elements such as transposons and insertion sequences can drive the evolution of plasmids and host. This study emphasises the importance of utilising long read sequencing technologies of plasmids and host strains at the earliest opportunity.

## Introduction

Clinical resistance to silver nitrate (AgNO_3_) in *Salmonella* Typhimurium was first reported by McHugh and colleagues in 1975 (Mchugh et al., 1975). The *S*. Typhimurium strain had been responsible for three deaths within the burns ward of Massachusetts General Hospital in 1973. Each of the three patients who died was receiving topical applications of 0.5% AgNO_3_ solution during treatment of severe burns. Blood- and wound-cultures all consistently produced AgNO_3_-resistant *S*. Typhimurium, which was also resistant to ampicillin, tetracycline, chloramphenicol, sulphonamides, streptomycin, and mercuric chloride (HgCl_2_). Conjugation studies demonstrated transmission of metal (AgNO_3_/HgCl_2_) and antibiotic resistances to other *Salmonella* spp. and to *Escherichia coli* K-12 J53. The plasmid responsible was designated pMG101 and is historically important as Mchugh et al. (1975) gave the first description of transmissible AgNO_3_-resistance in the clinic. Although the original *S*. Typhimurium isolate has not been the focal point of further published research (and may no longer exist in available culture collections), the *E. coli* J53/pMG101 transconjugant strain has been studied further. Pulse-field gel electrophoresis (PFGE) of pMG101 DNA indicated an approximate size of 180Kb, and a novel silver resistance operon was identified (Gupta et al., 1999). Assignment of pMG101 to the IncHI group and comparison of AgNO_3_-resistance encoding genes of the *sil* operon with other plasmids has given insights into the distribution of similar elements in IncH plasmids (Gupta et al., 2001). A whole genome sequence of a stock of *E. coli* J53/pMG101 (NCTC 50110) obtained from NCTC (National Collection of Type Cultures, United Kingdom) using Illumina MiSeq technology (Accession No. ASRI00000000) was previously published (Randall et al., 2015). The calculated size of pMG101 from the published short read sequence assembly was estimated as 183.5Kb, which agreed with the S1-endonuclease PFGE size estimate for pMG101 of 180Kb (Gupta et al., 1999). The sequence data showed evidence of Tn*7*-mediated transposition of the silver (*sil*) and copper (*pco*) resistance operons as a single unit (Tn*7*/*sil*/*pco*) into the *E. coli* J53 chromosome (Randall et al., 2015). The antimicrobial resistance gene (ARG) and metal resistance gene (MRG) content of pMG101 was however, congruent with the observed phenotypic resistances originally reported by Mchugh et al. (1975). A class D β-lactamase (bla-_OXA-1_), tetracycline resistance (*tetA*), chloramphenicol acetyltransferase (*catA*), sulphonamide resistance (*folP*), and the mercury resistance (*mer*) operon were all reported as being present in pMG101 (Randall et al., 2015).

IncHI plasmids constitute a diverse collection of large (75–400Kb), self-transmissible, low-copy number mobile genetic elements commonly found in Gram negative *Enterobacteriaceae* (Rozwandowicz et al., 2018). Molecular typing of IncHI plasmids provides discrimination between subgroups IncHI1, IncHI2, and IncHI3. IncHI2 plasmids are prevalent in *Enterobacteriaceae* associated with causing disease in humans and animals. As such, plasmids from this incompatibility group constitute a threat to public health and animal welfare. Of significance, *Salmonella* spp., *E. coli*, *Klebsiella pneumoniae*, and *Enterobacter cloacae* have all been identified as carriers of IncHI2 plasmids (Wong et al., 2016; Matamoros et al., 2017; Zhao et al., 2018; Chaudhry et al., 2020). Gene operons encoding metal resistances, such as tellurium (*terZABCDE*/*Y3Y2XY1W*), mercury (*merRTPCADE*), copper (*pcoABCDRSE*), and silver (*silESRCBAP*) are also common-place in this plasmid family (Fang et al., 2016). Co-occurrence of MRGs with multiple ARGs is widely reported (Pal et al., 2015; Li et al., 2017), and a recent study has highlighted the diverse array of ARGs encoded in IncHI2 plasmids isolated from poultry, ducks, and pigs in China (Fang et al., 2016). Extended spectrum β-lactamases (ESBL), plasmid-mediated quinolone resistance determinants (PMQRs), and Tn*7*/*pco*/*sil* transposition units are frequently seen in IncHI plasmids (Fang et al., 2016; Wang et al., 2017; Billman-Jacobe et al., 2018; Branger et al., 2018). Further expansion of the genetic content of IncHI plasmids can be driven *via* the acquisition of IncFI, IncFII, and IncN plasmids co-residing in the same bacterial cell. Fusion between two separate incompatibility groups (e.g., IncHI2/IncFII) may provide unique opportunities for plasmid host-range expansion allowing dissemination into novel hosts.

Given the historical interest of pMG101 as an archetype of AgNO_3_-resistance within the clinic and its relevance to current trends in AMR research, this mobile genetic element represents an important aspect of IncHI plasmid biology. Technological developments in long-read DNA sequencing provide a useful tool for resolving features of bacterial chromosomes and plasmids. Resolution of genomic repeat regions, multi-copy insertion sequences, and transposable elements is quite often beyond the reach of short-read sequencing platforms (Rhoads and Au, 2015), but long-read sequencing can overcome such issues, and in order to build upon the previously published sequence data of pMG101 (Randall et al., 2015) we decided to reanalyze this important mobile genetic element. Due to the advances in long-read and hybrid assembly technologies, it is possible to resolve some of these repeat regions. The technology may also be able to allow identification of genomic rearrangements and changes in the strain genome after longer term storage. The changes after long term storage would be expected to be small but support the theory that strains will have the capacity to undergo recombination after years in culture. Genome sequencing of microorganisms offers a snapshot to the genome and plasmidome as part of a continuous landscape (Hobman et al., 2007).

Using two separate stocks of *E. coli* J53/pMG101 obtained from different laboratories, PacBio sequencing of both strains and plasmids was used to provide complete closed-circular chromosomal and plasmid DNA sequences. Data obtained provided confirmation of previously published work regarding pMG101 such as ARG content and Tn*7*/*pco*/*sil* transposition into the *E. coli* chromosome. However, previously undescribed features of pMG101 including two distinct genotypes were identified from the sequence data, and further confirmed *via* S1-endonuclease PFGE. Here, we present analysis of each isoform – pMG101-A is a 383Kb IncHI2/IncFI/IncFII plasmid, whereas pMG101-B is a 154Kb IncFI/IncFII plasmid. Evidence obtained in the resequencing of these two plasmids, has allowed us to suggest a model for the transposition of the Tn*7*/*pco*/*sil* module into the chromosome of *E. coli* K-12 J53.

Given the medical relevance of silver as an antimicrobial able to enhance the effects of antibiotics, surveillance of resistance operons such as *sil* in a Tn*7* transposable element across conjugative plasmids and novel hosts in lab and environmental hosts is of significance (Zhou et al., 2015).

## Materials and Methods

### Bacterial Strains and Growth Conditions

Two separate stocks of *E. coli* J53 harbouring pMG101 were obtained from Anne Summers, University of Georgia, United States (pMG101-A) and Simon Silver, University of Chicago, United States (pMG101-B). Frozen (−80°C) glycerol stocks of both strains were used to inoculate LB agar plates prior to incubation overnight at 37°C. *Escherichia coli* J53 carrying a chromosomal insertion of *mrfp1* and *kan^r^* was used as a recipient in conjugation experiments (J53 RFP). For liquid growth, a single well-isolated colony was used to inoculate LB broth prior to overnight incubation at 37°C with shaking, unless stated otherwise.

### Pulse Field Gel Electrophoresis

Pulse-field gel electrophoresis was used to determine the sizes of the two plasmids pMG101-A and pMG101-B (Hooton et al., 2011). A single colony of *E. coli* J53 pMG101-A and *E. coli* J53 pMG101-B were inoculated in to separate 10ml Miller’s lysogeny broth -LB (Merck, United Kingdom). Broth cultures were incubated at 25°C for 48h and shaking at 180rpm. Following incubation cultures were centrifuged at 4,400×*g* for 5min, the supernatant was decanted, pellets were re-suspended in 1ml LB Broth, and 55μl of cell suspension was transferred to a 1.5ml micro-centrifuge tube. For PFGE plug preparation, 5μl of 20mg/ml proteinase K (Sigma Aldrich, United Kingdom) was added followed by mixing 50μl of molten 1.2% PFGE grade agarose (Biorad, United States) in TE [10mM Tris-HCl (pH 7.5), 1mM EDTA] buffer. The suspension was mixed thoroughly and then set in PFGE plug moulds (Biorad, United States). The plugs were then incubated overnight at 55°C shaking at 300rpm in 1ml lysis buffer [50mM Tris-HCl (pH 8), 50mM EDTA, 1% *N*-lauroyl sarcosine, and 100μg/ml proteinase K]. Plugs were then washed three times for 1h in 1ml wash buffer [20mM Tris-HCl (pH 8), 50mM EDTA] at 55°C shaking at 300rpm. A 3mm slice of each plug was then loaded to a 1% PFGE grade agarose gel (100ml 1 X TAE buffer) along with PFGE lambda ladder (New England Biolabs, United States). The lanes were sealed with 1% PFGE grade agarose. A CHEF-DR II system was used for the run with a 10–30s switch time over 18h at 6V/cm, and 1 X TAE running buffer was circulated at 14°C. The gel was then stained using ethidium bromide (1μg/ml) and imaged using a Biorad ChemiDoc MP System (BioRad, United States).

### Conjugation of pMG101-A and pMG101-B

A single colony of *E. coli* J53 pMG101-A, *E. coli* J53 pMG101-B and *E. coli* J53 RFP were inoculated in to separate 20ml Miller’s LB and incubated at 25°C with shaking until an OD_600nm_ 1.0 was reached. About 1ml of each culture was then pelleted at 16,000g for 1min, the supernatant was removed before being resuspended and washed in 1ml maximum recovery diluent – MRD (Oxoid, United Kingdom). The wash step was repeated once more. Around 50μl of each culture containing pMG101 was added to 50μlJ53 RFP, mixed thoroughly by pipetting, and deposited on to non-selective LB agar plates and incubated at 25°C for 48h. The lawn of growth was then scraped off each plate and resuspended in 1ml MRD and vortexed thoroughly to end mating. The cells were then pelleted at 16,000g for 1min and supernatant removed and resuspended in 1ml MRD. The wash step was repeated once. The OD_600nm_ was then measured and adjusted to 1.0 with MRD. About 10-fold dilutions of the cell suspension were then made and spread plated in triplicate onto LB agar supplemented with 25μg/ml HgCl_2_ and incubated at 25°C for 48h. Successful transconjugants, that produced red pigment, and were able to grow in the presence of HgCl_2_, were counted. Transconjugants were then selected at random, and patch plated to LB agar supplemented with 25μg/ml HgCl_2_ and non-selective LB agar to confirm conjugation.

### DNA Extraction

Genomic DNA was extracted from overnight *E. coli* J53 LB broth cultures using a GenElute™ Bacterial Genomic DNA Kit (Sigma Aldrich, United Kingdom) as per manufacturer’s instructions. DNA integrity was checked by electrophoresing 5μl of each preparation in a 1% agarose gel with Quick-Load® 1Kb DNA ladder (New England Biolabs, United Kingdom) as a molecular weight marker. Genomic DNA extracts were quantified using an Invitrogen™ Qubit™ 4 Fluorometer (Fisher Scientific, United Kingdom) to determine overall yield.

### Pacific Biosciences Library Preparation and Sequencing

Genomic DNA samples were purified with 1x cleaned AMPure beads (Agencourt, United Kingdom), and the quantity and quality was assessed using a Nanodrop spectrophotometer and Qubit assay. Additionally, the Fragment Analyser (Agilent, United Kingdom; using a high sensitivity genomic kit) was used to determine the average size of the DNA and the extent of degradation. This procedure was also used at the steps indicated below to determine average fragment size of the DNA. One microgram of DNA for each sample was sheared to approximately 10Kb using a Covaris G tube (Beckman Coulter, United Kingdom), as per manufacturer’s instruction. The average size was checked using a Fragment Analyser (Agilent, United Kingdom). Samples were bead purified and subjected to a DNA damage repair reaction at 37°C for 1h followed by an end repair reaction at 25°C for 5min. Bead cleaning was performed as described above. Samples were individually ligated to barcoded blunt-end adapters overnight at 25°C. After a heat inactivation step at 65°C for 10min, the samples were pooled and treated with exonucleases 3 and 7 to remove any non-circular DNA. The library was size selected on Sage BluePippin (Beverly MA, United States) using a 0.75% cassette between 5 and 50Kb to remove any shorter material. The final library concentration was measured by Qubit assay, and DNA sizes were determined by Fragment analyser. Two SMRT cells were run for this DNA using diffusion loading and sequenced on the PacBio RS system. Libraries were sequenced using 600-min movies at the Centre for Genomic Research, University of Liverpool, Crown Street, Liverpool, L69 3BX, United Kingdom.

### Sequence Assembly and Bioinformatics

The resulting reads had adapters removed prior to the trimmed reads being assembled using the “canu” assembler tool, and subsequently polished using the “arrow” algorithm (Chin et al., 2013; Koren et al., 2017). Polished assemblies were quality checked for contiguity using “QUAST” with “busco” being employed to determine completeness of assemblies using Benchmarking Universal Single-Copy Orthologs (Gurevich et al., 2013; Simão et al., 2015). In this case, BUSCO V2 was used with the BUSCO proteobacteria database used as the reference. The assembled genomes for both *E. coli* K-12 J53 pMG101 variants were subsequently annotated using Prokka (Seemann, 2014) and screened for ARGs and MRGs using ResFinder (Bortolaia et al., 2020) and BacMet 2.0 database (Pal et al., 2014). Plasmid incompatibility groups were identified using PlasmidFinder (Carattoli et al., 2014).

Chromosome and plasmid sequences have been deposited at NCBI under Bioprojects PRJNA 701544 and PRJNA701546. Individual Genbank accession numbers host strain and plasmid sequences are *E. coli* J53/pMG101-A (JAFFIC010000001.1), pMG101-A (JAFFIC010000003.1), *E. coli* J53/pMG101-B/Tn7 insertion (CP070962), and pMG101-B (CP070963).

### Antimicrobial Susceptibility Testing

The two *E. coli* J53 pMG101 variants were tested for antibiotic susceptibility according to the Clinical and Laboratory Standards Institute standard protocol (CLSI, 2019). The following antibiotics were tested: ampicillin 10μg (AMP), amoxicillin-clavulanic acid 20 and 10μg (AMC), cefoxitin 30μg (FOX), ceftazidime 30μg (CAZ), cefpodoxime 10μg (CPD), aztreonam 30μg (ATM), imipenem 10μg (IPM), streptomycin 10μg (S10), tetracycline 30μg (T), ciprofloxacin 5μg (CIP), nalidixic Acid 30μg (NA), trimethoprim-sulfamethoxazole 1.25μg and 23.75μg (SXT), chloramphenicol 30μg (C), nitrofurantoin 300μg (F), and azithromycin 15μg (AZM). All discs were obtained from Pro-Lab Diagnostics (Birkenhead, United Kingdom). Antimicrobial metal resistance testing for HgCl_2_ (184μM) and AgNO_3_ (400μM) was carried out according to standard techniques (Yang et al., 2020).

## Results

### Pulsed Field Gel Electrophoresis

Imaging of the plasmids extracted from the two J53 strains by PFGE showed a significant difference in size between the two large, low copy number plasmids. pMG101-A was identified as approximately 380Kb, more than double the size of pMG101-B at approximately 170Kb. Due to the size and the copy number of the plasmids, the band intensity was significantly reduced although was still clearly visible (Supplementary Figure S1, Supplementary data).

### Sequencing of IncHI2/IncFI/II Plasmid pMG101-A and IncFI/FII Plasmid pMG101-B

**Figure 1:**
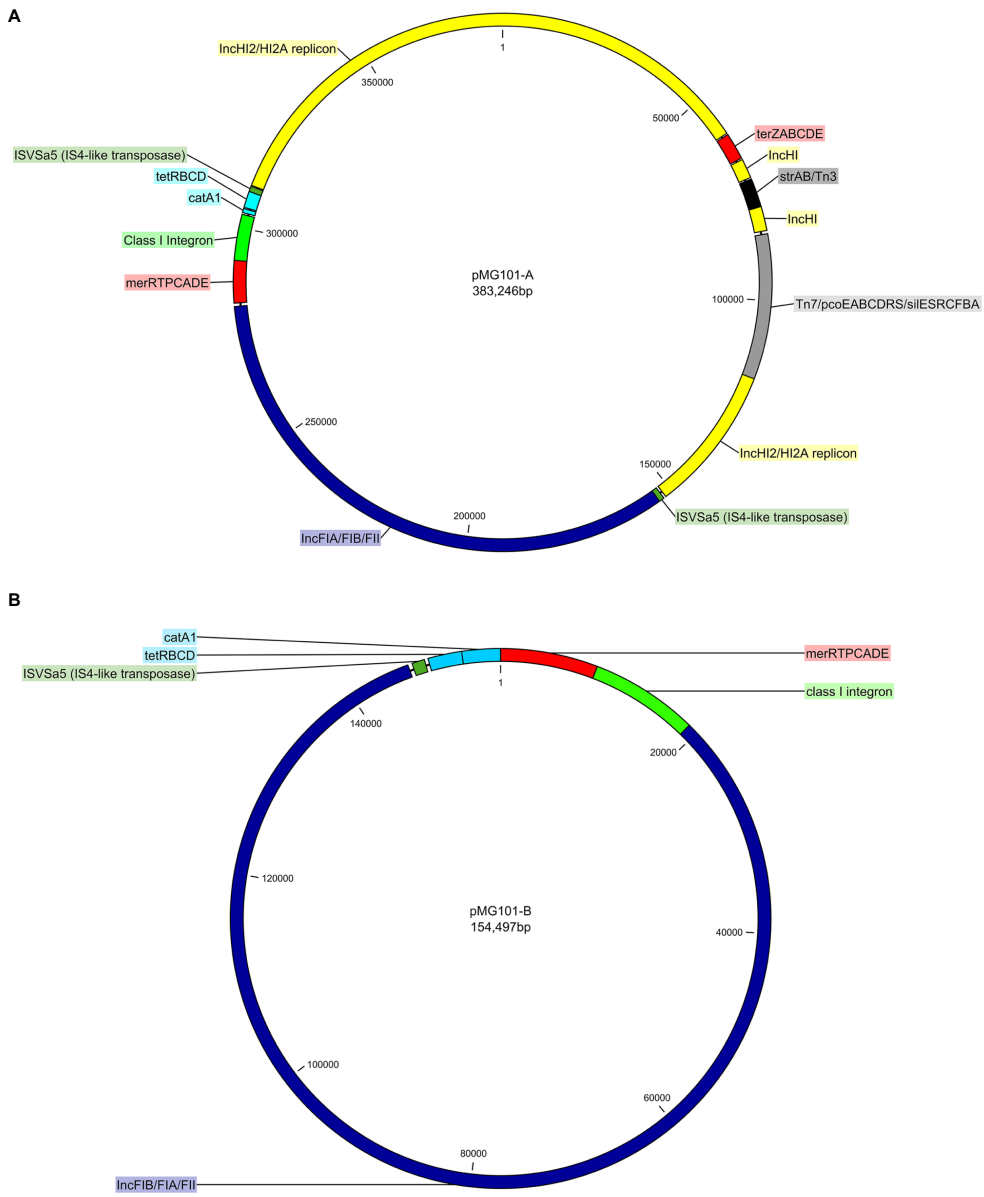
**(A)** Circular genetic map of IncH/IncF plasmid pMG101-A – the IncHI2/HI2A replicon (yellow), IncFIA/FIB/FII component (blue), Tn*7*/*pco*/*sil* mobile unit (grey), *ter* and *mer* metal resistance operons (red), Tn*3*/*strAB* antimicrobial resistance gene (ARGs; black), *tetBCD*/*catA1* (light blue), class I integron (light green), and two copies (dark green) of ISVSa5 (IS4-like transposase) are all highlighted. **(B)** Circular genetic map of IncF plasmid pMG101-B – the IncFIA/FIB/FII backbone of pMG101-B (blue), class I integron (light green), *tetBCD*/*catA1* (light blue), *mer* operon (red), and a single copy (dark green) of ISVSa5 (IS4-like transposase).

Previously, we had sequenced the ~380Kb pMG101-A plasmid using Illumina MiSeq. Assembly of short reads produced an approximately 300Kb plasmid sequence, albeit fragmented in 27 contigs ranging from 120 to 34 and 220bp in length (Unpublished data). To improve assembly of pMG101-A, we utilised long-read PacBio sequencing. Assembly of 10Kb PacBio reads yielded a single closed-circular 383,246bp dsDNA molecule representative of pMG101-A. The size is in agreement with S1-endonuclease PFGE analysis of cultures of the *E. coli* J53 strain harbouring pMG101-A (Figure 1). Analysis of the nucleotide composition revealed a GC content of 47.8%. Identification of plasmid replicons in pMG101-A was achieved using the online tool PlasmidFinder (Carattoli et al., 2014). Five separate replicons were identified in pMG101-A representing incompatibility groups IncH and IncF (Tables 1, 2). Several features haracteristic of IncH plasmids are apparent in pMG101-A (Figure 1A) including the tellurium resistance operon (*terZABCDE*/*terY3Y2XY1W*), *smr* loci 0018/0199, IncH conjugal transfer (*tra1/tra2*) regions, and plasmid replicons of the IncHI2/IncHI2A sequence types (Carattoli et al., 2014). A single 4,634,108bp contig for the host strain *E. coli* K-12 J53 harbouring pMG101-A was obtained.

**Table 1:** Plasmid replicon typing of pMG101-A and pMG101-B.

Locus	pMG101-A		pMG101-B	
	Start	End	Start	End
IncFIB	182,235	182,916	117,399	118,080
IncFIA	215,984	216,371	83,944	84,331
IncFII	276,913	277,173	23,142	23,402
IncHI2	335,674	336,000	–	–
IncHI2A	350,868	351,497	–	–

**Table 2:** Plasmid double locus sequence typing of pMG101-A.

Locus	Allele	Length	Start	End
FIA	1	384	215,964	216,347
FIA	6	329	216,019	216,347
FIB	1	373	182,494	182,866
FII	1	157	276,999	277,155
smr0018	2	330	369,393	369,722
smr0199	2	460	133,673	134,132

As was found with pMG101-A, Illumina sequencing of plasmid pMG101-B yielded a fragmented assembly (Unpublished data) containing gaps, unresolvable small contigs etc. The *pco*/*sil* genes were noticeably absent from the plasmid assembly. We used PacBio sequencing of the J53 pMG101-B strain to understand the biology of the plasmid and to confirm chromosomal integration of the *pco*/*sil* operon. A single closed-circular 154,497bp dsDNA molecule representative of pMG101-B was assembled from PacBio reads as described above (Figure 1B). A single 4,683,867bp contig representative of the host *E. coli* K12 J53/Tn*7* insertion harbouring pMG101-B was also assembled. Analysis of the nucleotide content of pMG101-B indicates a higher GC content (53.28%) compared to pMG101-A (47.8% GC). As can be seen from Table 1, pMG101-B is an IncF plasmid containing replicon-types IncFIA, IncFIB, and IncFII.

### pMG101-A and pMG101-B ARG and MRG Content

Resistance to toxic metals and antibiotics is encoded by MRGs and ARGs present in both pMG101-A and pMG101-B. Table 3 shows resistances, nucleotide positions, and associated mobile genetic elements (e.g., transposons/insertion sequences). Antibiotic sensitivity tests showed that resistance phenotypes of *E. coli* J53 carrying pMG101-A and pMG101-B were congruent with ARGs identified in the PacBio sequence assembly. Both J53 pMG101 strains were resistant to ampicillin, aminoglycosides, tetracycline, chloramphenicol, nalidixic acid, and sulphonamides, as well as AgNO_3_ and HgCl_2_. The discernible difference between the two variant pMG101 plasmids was *strAB*-encoded streptomycin resistance in pMG101-A, which was absent in pMG101-B.

**Table 3:** Locations of ARGs and metal resistance genes (MRGs) within pMG101-A and pMG101-B.

Gene(s)	Resistance	pMG101-A	pMG101-B
*sul1* ^*^	Sulphonamide	294,833–295,672	13,551–14,390
*qacE∆1* ^*^	Quaternary ammonium compounds	295,666–296,013	14,384–14,731
*aadA1* ^*^	Aminoglycoside	296,177–296,956	14,895–15,674
*bla*_OXA-1_ ^*^	β-lactam	297,081–297,956	15,799–16,674
*catA1*	Chloramphenicol	303,987–304,709	153,227–154,886
*tetRBCD*	Tetracycline	306,933–309,549	147,819–150,940
*strAB*	Aminoglycoside	71,740–73,489	–
*terZABCDE*	Tellurium	60,310–66,529	–
*merRTPCADE*	Mercury(II)	282,742–286,695	1,451–5,413
*silESRCFBA*	Silver(I)	95,329–104,678	4,560,169–4,572,614^chr^
*pcoEABCDRS*	Copper(II)	109,736–116,591	4,574,582–4,581,431^chr^

* *resistance gene belongs to gene cassette within Tn21-like integron*.

chr *location in the bacterial chromosome of E. coli K-12 J53*.

**Figure 2:**
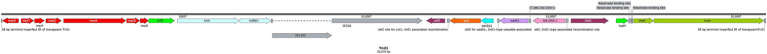
Genetic map of Tn*21* present in both pMG101-A and pMG101-B – the backbone consisting of the *mer* operon (red) and transposase genes (green). The insertion of the Tn*402*-like element containing Class I integron with cassette array encoding the following resistance genes: *bla_OXA-1_*, *aadA1*, *qacE∆1*, and *sul1*. The Tn*21* mobile genetic element is flanked by two 38bp imperfect inverted repeats.

The shared ARG content of pMG101-A and pMG101-B consists of a classical Tn*21*-family mercury resistance operon and associated class I integron harbouring antibiotic resistance gene cassettes (Figure 2). The *mer* operon (*merRTPCADE*) and class I integron structure is 20,676bp in length and forms a composite transposable element of the Tn*3* family (Tn*21* subgroup). Both sides of the transposon are flanked by 38bp imperfect inverted repeats (IRs) with the left-IR positioned immediately upstream of *merR* and the right-IR immediately downstream of the Tn*21* transposon. Moving inwards from the *mer* operon, a further set of embedded transposable elements is seen. A Mu-like transposon (*tniA*/*tniB*) and an IS*26*-like element (*istA*/*istB*) constitute the region between the *mer* operon and the class I integron. The 3'-conserved segment of the class I integron contains a typical arrangement of *sul1*/*qacE* that encodes resistance to sulphonamides and quaternary ammonium compounds, respectively. Resistance to aminoglycoside antibiotics is encoded by the *aadA1* gene cassette and is located immediately downstream of *sul1*. A class D β-lactamase gene, *bla*_OXA-1_, a progenitor of the well-established and studied oxacillinases is the next antibiotic resistance gene cassette in the array of the class I integron. This suggests *bla*_OXA-1_ was the most recently captured cassette in the integron cassette array. Finally, the diagnostic feature of the class I integron, the *intI1* integrase, is located at the 5'-region. Immediately downstream of *intI1* is the previously described Tn*21* family transposon unit. Three genes encoding the modulation function (*tnpM*), a resolvase (*tnpR*), and the transposase (*tnpA*) form the Tn*21* transposable element. This region is followed by the right-IR, which defines the end of the composite transposon. Immediately, downstream of the Tn*21* unit is a chloramphenicol acetyltransferase (*catA1*) gene that confers resistance to phenicol antibiotics. IS*1*-like transposon genes (*insA*/*insB*) and a tetracycline resistance transposable unit (*tetRBCD*) are the final ARGs in this multidrug and metal resistance region.

### IS-Mediated Fusion of IncHI/IncF pMG101-A

As discussed previously, the 383,246bp pMG101-A is composed of IncH1/H12A/FIA/FII/FIB plasmid sequences. Based on sequence analysis and mapping of insertion sequences and transposable elements, it is possible to hypothesise a chain of events leading to fusion of the various Inc. plasmid regions. Downstream of conjugal transfer region 2 of the IncH1/H12A element is an ISVSa5 (IS*4*-like transposon) that defines the beginning of the IncF region. Approximately, 157Kb further downstream is a further ISVSa5 transposon that delineates the end of the IncF plasmid sequence. This ISVSa5 element is located immediately downstream of *tetD*. Due to the presence of these elements, at the junctions of the IncH components of the plasmid, it is possible that the integration event has been driven by ISVSa5-mediated recombination. An interesting feature of the IncF region is its similarity to a chromosomal genomic island (GI-DT12) previously observed in *S*. Typhimurium T000240. Chromosomal insertion of this large genomic island is reported as being mediated by IS1-like transposable elements (Izumiya et al., 2011). The major differences between GI-DT12 and the homologous regions found in both pMG101 variants are the presence of IncF replicons in the plasmid nucleotide sequences. The two copies of ISVSa5 that flank the IncF region of pMG101-A may have arisen following a duplication event that occurred during recombination. Analysis of pMG101-B shows that there is only a single copy of ISVSa5 present in this plasmid, which is positioned downstream of *tetD*.

### Factors Influencing Conjugal Transfer of pMG101-A

Multiple attempts to transfer pMG101-A *via* conjugation into different *E. coli* recipient strains at 25 and 37°C were undetected, whereas pMG101-B transferred into *E. coli* recipients at 25°C. The pMG101-B plasmid was transferred into the recipient strain *E. coli* K-12 J53 RFP kan^r^ at a frequency of 1.25×10^−3^ conjugants/ml. Analysis of the conjugal transfer regions of pMG101-A highlighted the presence of an ISEc27 element that had integrated into *orf009* of the transfer region 2 (*tra2*). It has recently been shown that *orf009* (herein designated *rsp*) encodes an essential component of the conjugation apparatus – the RSP/R27-secreted protein (Huttener et al., 2019), suggestive that ISEc27 has inactivated the transfer ability of pMG101-A. ISEc27 is found as multiple copies in both *E. coli* K12 J53 chromosomes with a total of nine in the strain harbouring pMG101-A (plus one insertion in pMG101-A *rsp*), and seven copies in *E. coli* J53 pMG101-B. No insertions of ISEc27 were identified in pMG101-B. Expansion of mobile genetic elements such as ISEc27 in long-term stocks derived from strains such as *E. coli* K12 J53 should be monitored for such events.

### Tn*7*-Mediated *sil*/*pco* Chromosomal Integration

**Figure 3:**
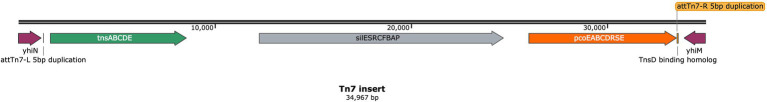
Tn*7*-mediated transposition of metal resistance operons *pco* and *sil* into *Escherichia coli* J53 chromosome – the transposition event has resulted in insertion of the mobile unit between *yhiN* and *yhiM*.

As previously reported the NCTC stock of *E. coli* J53 pMG101 strain carries a Tn7/*sil*/*pco* module chromosomally, conferring resistance to copper- and silver-containing compounds (Randall et al., 2015). In most instances, mobility of Tn*7*-elements is driven *via* Tn*7*-mediated transposition into the canonical *att*Tn*7* site located at the 3'-end of *glmS* in the *E. coli* chromosome. Analysis of the Tn*7*/*sil*/*pco* region in pMG101-A shows that the complete module (*tnsABCDE*/*silESRCFBA*/*pcoEABCDRS*) is located in the plasmid DNA assembly – specifically within the IncHI region of the plasmid. IncHI plasmids and their relatives are recognised as providing an environmental reservoir of MRGs, including *pco*/*sil* within the family *Enterobacteriaceae* (Gupta et al., 2001; Fang et al., 2016). For pMG101-B, the Tn*7*/*sil*/*pco* module has transposed into the chromosome of the *E. coli* J53 host. The transposition event has mediated the integration of Tn*7*/*sil*/*pco* in between *yhiN* (NAD[P]/FAD-dependent oxidoreductase) and *yhiM* (inner membrane protein; Figure 3). The overall effect of this is that *yhiN*/*yhiM* is now split by the Tn*7*/*sil*/*pco* module such that the two genes are separated by a genomic island of approximately 33.7Kb in length. The absence of Tn*7*/*sil*/*pco* in the plasmid sequence of pMG101-B is also coupled to the complete absence of all the recognisable features of the IncH component of pMG101-A. Analysis of *glmS* in *E. coli* J53/pMG101-B shows that the site is unoccupied with respect to the presence of Tn*7*-like elements. The use of alternate insertion sites in the *E. coli* chromosome for the Tn7/*sil*/*pco* that are not the canonical *att*Tn*7* has also been observed in pig *E. coli* isolates (Chalmers et al., 2018).

## Discussion

Observable differences in antibiotic resistances and PFGE size profiles of plasmid pMG101 between what was expected and previously reported data (Randall et al., 2015) drove us to investigate the underlying mechanisms driving these changes. Previously, published Illumina MiSeq data for pMG101 hinted at some unusual biology but the fragmented assembly (Randall et al., 2015) and difficulties faced when resolving repeat sequences (Cahill et al., 2010) suggested a long-read sequencing approach may improve our current understanding of pMG101. Therefore, we applied PacBio long-read sequencing in order to resolve the important features of pMG101, gain complete closure of the circular genetic element, and determine any alterations to the host *E. coli* K12 J53 chromosome. Working from two separately obtained stocks of pMG101, it was possible to show evidence of plasmid recombination between IncH and IncF plasmids (pMG101-A and pMG101-B), Tn*7*-mediated transposition of MRGs into a non-canonical *att*Tn*7* site in the chromosome of *E. coli* J53 (pMG101-B), and loss of mobilisation (pMG101-A). The transposition event of pMG101-B Tn7/*sil*/*pco* is consistent with other reports that the Tn*7* element can insert into the chromosome of other *E. coli* strains at non-canonical *att*Tn*7* sites (Chalmers et al., 2018). Transposition of the Tn*7* element resulted in the host strain gaining chromosomal resistance to silver and copper in the form of *silESRCFBAGP* and *pcoEABCDRS*, which form with the Tn*7* transposition machinery – a recognised, likely ancient, and widely distributed, mobile genetic element (Hao et al., 2015; Hobman and Crossman, 2015; Staehlin et al., 2016). The use of this *att*Tn*7* suggests that the chromosomal insertion occurred *via* the TnsABC+D rather than the TnsABC+E system. Whilst both of the mechanisms are able to induce transposition to the chromosome, TnsABC+D promotes insertion by “cut and paste” mechanism to the specific attTn*7* sites (5'-CCCGC-3'), which is duplicated and normally 23 bases from the C-terminal end of the *glmS* gene (Waddell and Craig, 1989; Bainton et al., 1993; Mitra et al., 2010). In the case of pMG101-B, the same *att*Tn*7* site is observed between the *yhiN* and *M* genes in the *E. coli* J53 chromosome. Tn*7* and Tn*7*-like elements undergo chromosomal insertion by inserting downstream (3') adjacent to *glmS* within the host chromosome, as it is considered a safe site that does not interfere with host cell function and is therefore more likely to propagate to the daughter cells (Peters and Craig, 2001). The mechanism for insertion has been exploited and used to produce high copy number expression vectors such as pRM2, a pUC18 backbone with a Tn*7* insertion site also presenting resistance to ampicillin (Mckown et al., 1988).

From the assembled data, it is seen here that from the original location in pMG101-B, Tn*7*/*pco/sil* has inserted using this mechanism between *yhiN* and *yhiM*. Chromosomal insertion of Tn*7* is documented to be less frequent than transposition to conjugal plasmids. In this case, pMG101-A has lost mobilisation ability due to *orf009*, a part of the *tra* genes needed for conjugative plasmid transfer, having an integrative and conjugative element (ICE) inserting in the middle of it and separating it into two parts. Over time, it is possible that the loss of conjugative ability of the plasmid may have increased the likelihood that the TnsABC+D mechanism for chromosomal insertion of the Tn*7* element as a means to make sure it is passed onto daughter cells (Craig, 1996). This hypothesis is in agreement with the reported function of TnsC, which may evaluate insertion target sites for *attTn7* sites acting as a regulator for the TnsD and E proteins. TnsD then binds to a consensus sequence allowing for insertion of the Tn*7* (Hauer and Shapiro, 1984). While a similar chain of events has allowed pMG101-B/Tn*7* to integrate at an alternative chromosomal locus, limited sequence homology between *attTn7*/*glmS* and the insertion site of *yhiNM* is observed.

Insertion of mobile genetic elements to host DNA may be advantageous, but equally may have less favourable implications. Transmissibility of IncHI plasmids from host to recipient cells is determined by the presence of specific transfer regions – Tra1 and Tra2. In this instance, the ISEc27 element that has undergone expansion in numbers in the *E. coli* K12 J53 (pMG101-A) host strain is inserted on the opposite strand of Tra2 *rsp*, is transcribed in the opposite direction to all other genes encoded in the gene operon and may well also be exerting polar effects on genes downstream that are recognised as being essential for conjugal transfer (*trhFWUN*). For IncHI plasmids, R27 isolated from *S*. Typhi and prototypical founder for the IncHI1 plasmid group remains one of the best studied plasmids of this type (Sherburne et al., 2000; Lawley et al., 2003; Huttener et al., 2019; Luque et al., 2019). In *S*. Typhimurium SL1344, the 155.4kDa RSP was initially identified in cell-free supernatants and found to be expressed in a temperature-dependent manner (optimal expression 25°C). RSP is exported to the extracellular surface *via* the plasmid-encoded type IV secretion system in a *trhC*-dependent manner. Externally RSP has been identified as primarily interacting with flagella, which consequently reduces cell-motility, an initial step towards bacterial conjugation. However, parallel studies with non-flagellated cells also indicate other external cellular components may interact with RSP to reduce motility (Huttener et al., 2019). Disruption of essential transfer genes such as *rsp* would therefore likely have a negative effect on conjugation ability as observed for *E. coli* K12 J53 (pMG101-A).

From the two *E. coli* J53 pMG101 cell stocks we analysed and the similarity of one stock (of pMG101-B) to the NCTC50110 stock sequenced by Randall et al. (2015), it is quite clear that there are some significant variations between the two plasmids and host strains. The fusion of a whole plasmid replicon larger than the first reported analysis of pMG101 in one stock (Gupta et al., 2001) and loss of transmissibility resulting in chromosomal insertion of the Tn*7*/*pco*/*sil* mobile element in the other suggest that bacterial evolution during long term storage and use in laboratories has occurred. This has broader, but unsurprising, implications for our understanding of isolates of plasmid containing strains, suggesting that in the real world these are likely to be transient forms, which can also evolve in the laboratory. Whilst sequence analysis demonstrates what the plasmid structure is, what the sequence of events that led up to these significant differences in pMG101 was, is less easy to reconstruct in a timeline. Over 20years passed between the original reports of transfer of pMG101 into *E. coli* K-12 and reports of the size of the plasmid by PFGE. It is clear that at some point in the history of pMG101, two plasmids fused, one of which carried a Tn*7/sil/pco* mobile genetic element and the other of which carried SGI11. It is also clear that the Tn*7/sil/pco* element transposed into a non-standard *attTn7* site in the *E. coli* chromosome. As there is now cheap and readily accessible long-read sequencing available for bacterial strains, we argue that researchers should take advantage of this technology for early sequencing of minimally manipulated isolates and use sequencing as a tool to regularly check strain integrity.

## Data Availability Statement

The datasets presented in this study can be found in online repositories. The names of the repository/repositories and accession number(s) can be found in the article/Supplementary material.

## Author Contributions

SH, AM, DS, and JH conceived and designed the experiments. SH, AP, and C-GH performed experimental work. SH, AP, LC, C-GH, AM, and JH: bioinformatic analysis. SH, AP, KA, C-GH, LC, DS, AM, and JH: wrote the manuscript. All authors contributed to the article and approved the submitted version.

## Conflict of Interest

LC is the director of SequenceAnalysis.co.uk and was paid to perform this work.

The remaining authors declare that the research was conducted in the absence of any commercial or financial relationships that could be construed as a potential conflict of interest.

## Publisher’s Note

All claims expressed in this article are solely those of the authors and do not necessarily represent those of their affiliated organizations, or those of the publisher, the editors and the reviewers. Any product that may be evaluated in this article, or claim that may be made by its manufacturer, is not guaranteed or endorsed by the publisher.

## Supplementary Material

Supplementary Figure S1 PFGE analysis of S1-endonuclease digestion of pMG101-A (left image) and pMG101-B (right image).
