# Supplementary figures and images for: Laboratory Stock Variants of the Archetype Silver Resistance Plasmid pMG101 Demonstrate Plasmid Fusion, Loss of Transmissibility, and Transposition of Tn7/pco/sil Into the Host Chromosome
Source: Front Microbiol. 2021 Aug 19;12:723322. doi: 10.3389/fmicb.2021.723322 (PMC8417528; doi:10.3389/fmicb.2021.723322)

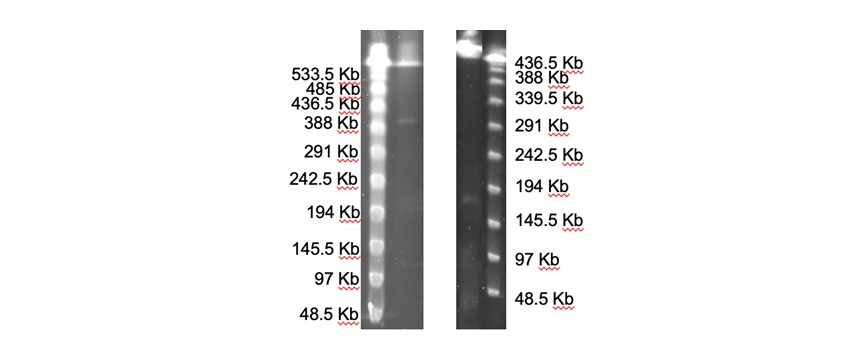

Supplement: Supplementary Figure S1 — PFGE analysis of S1-endonuclease digestion of pMG101-A (left image) and pMG101-B (right image). [file Image_1.JPEG]
